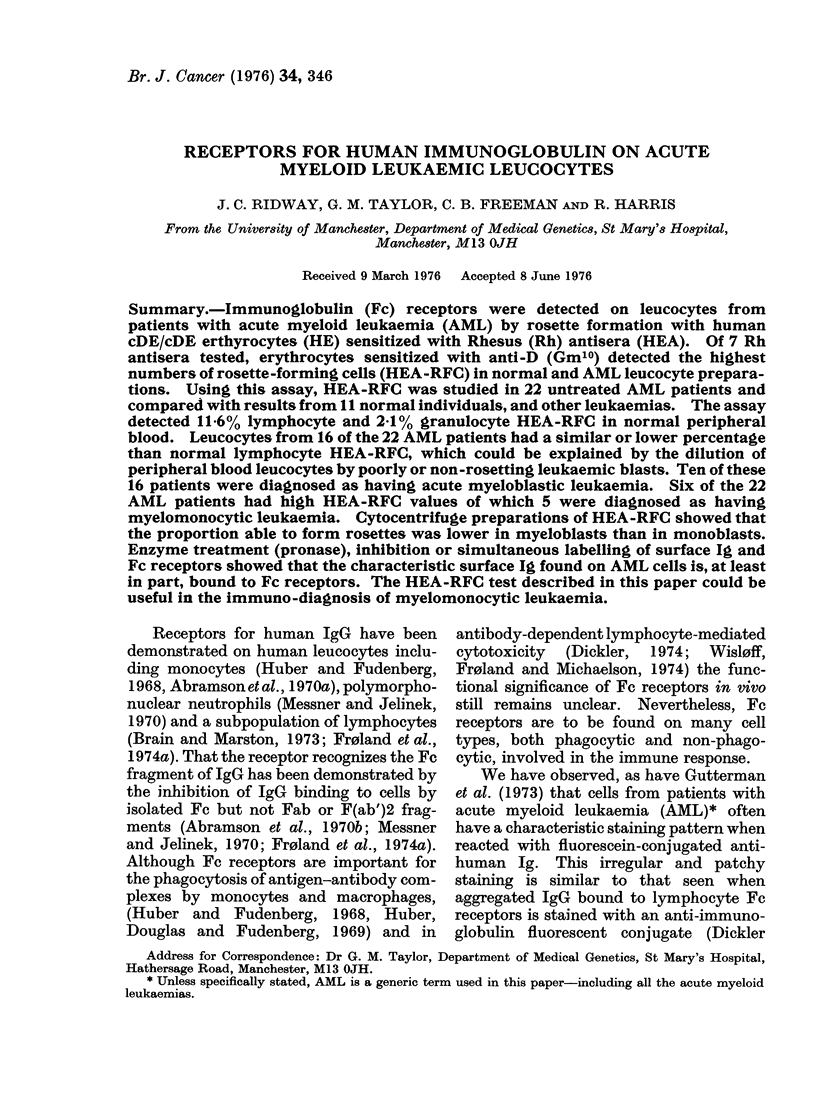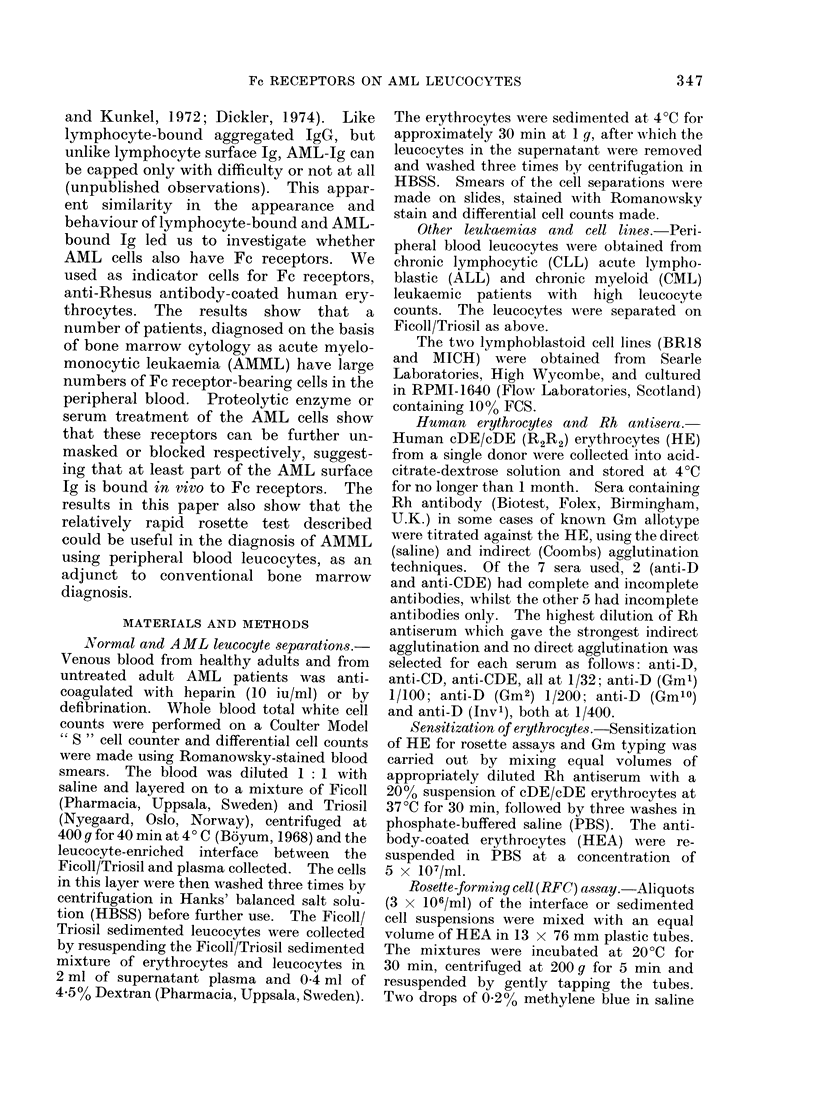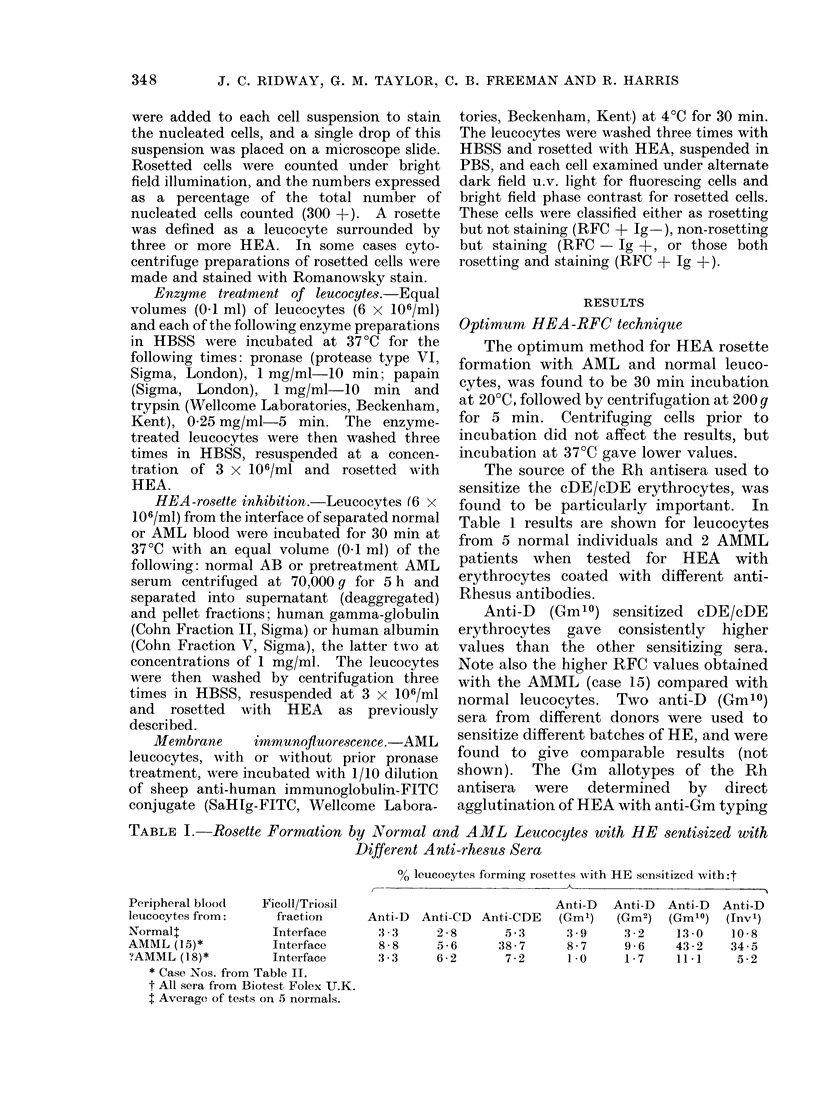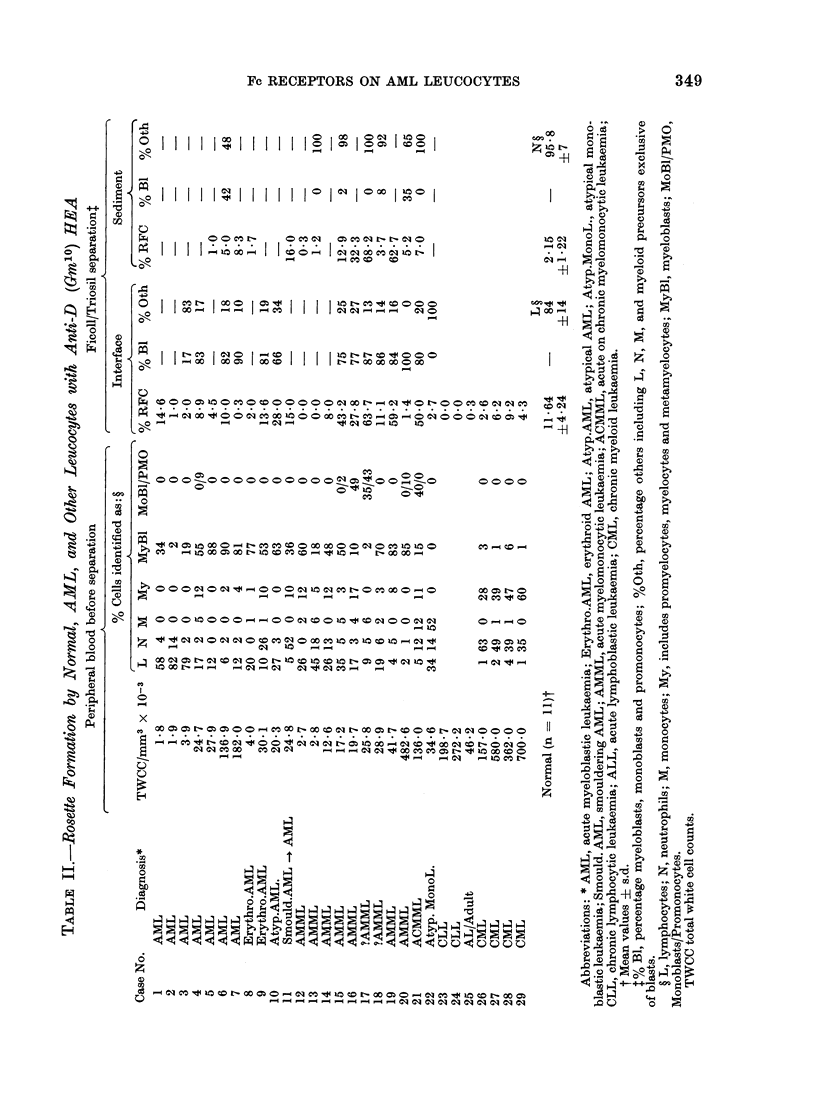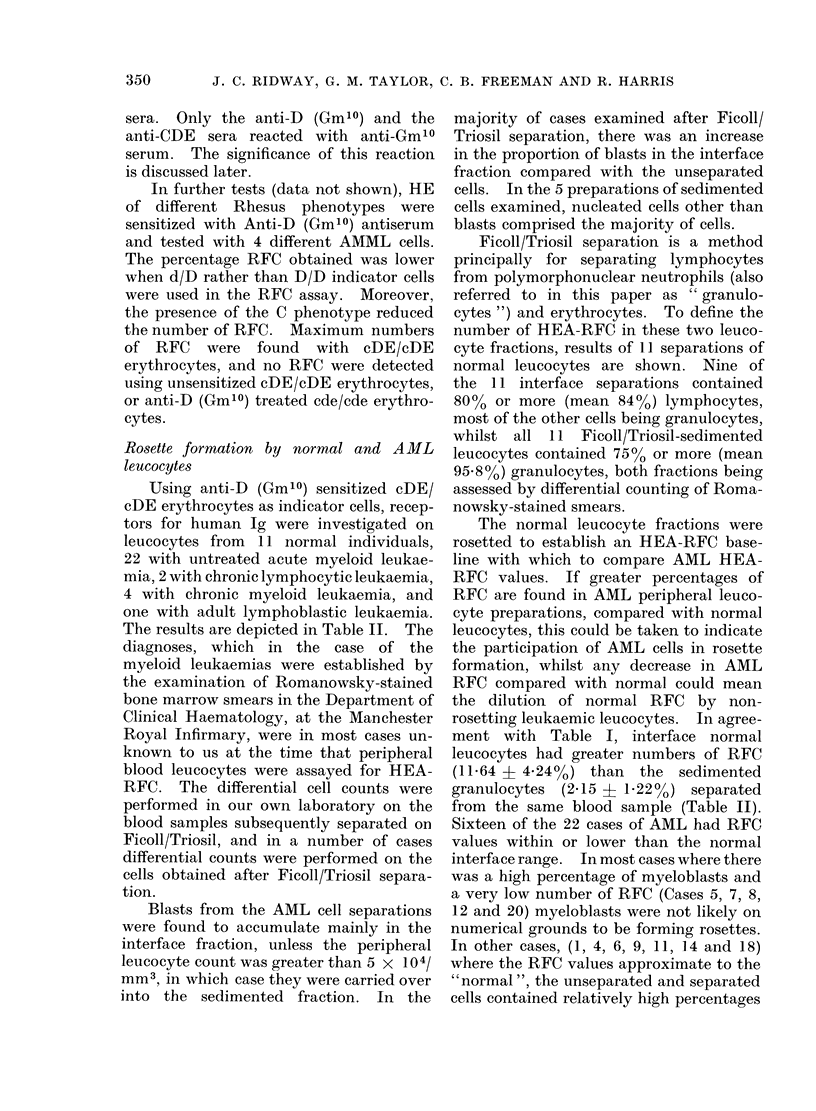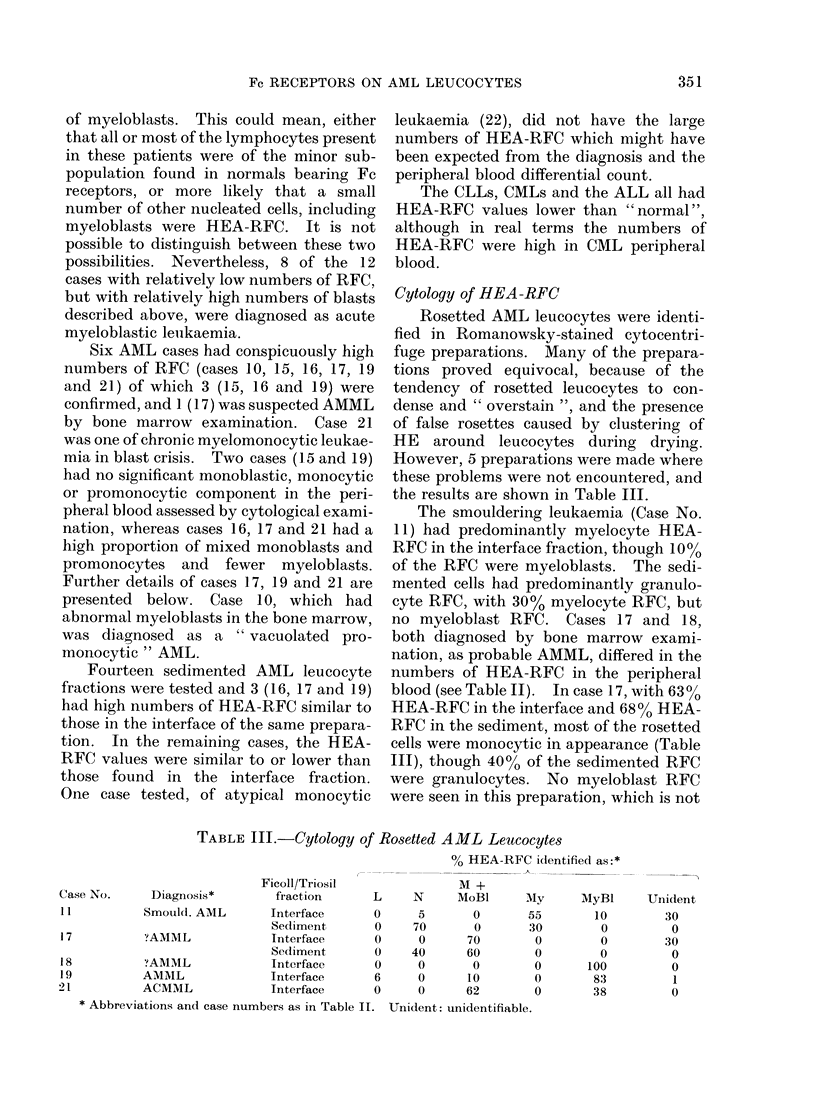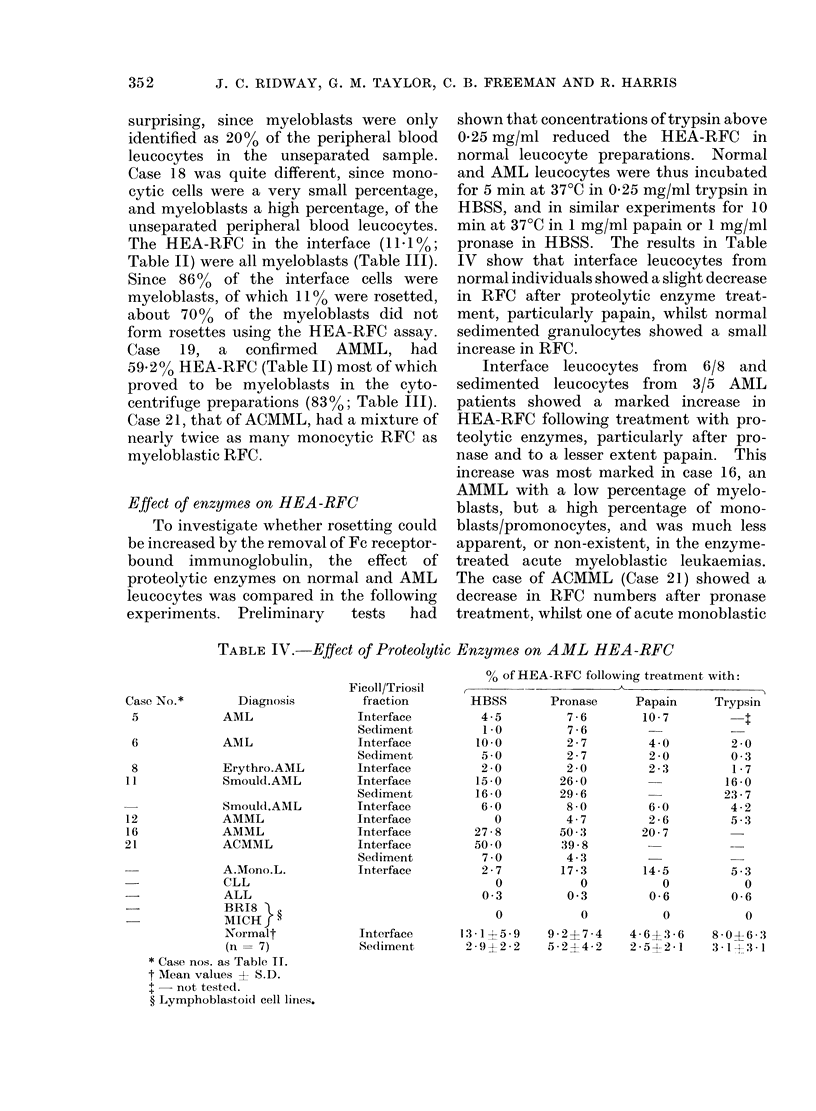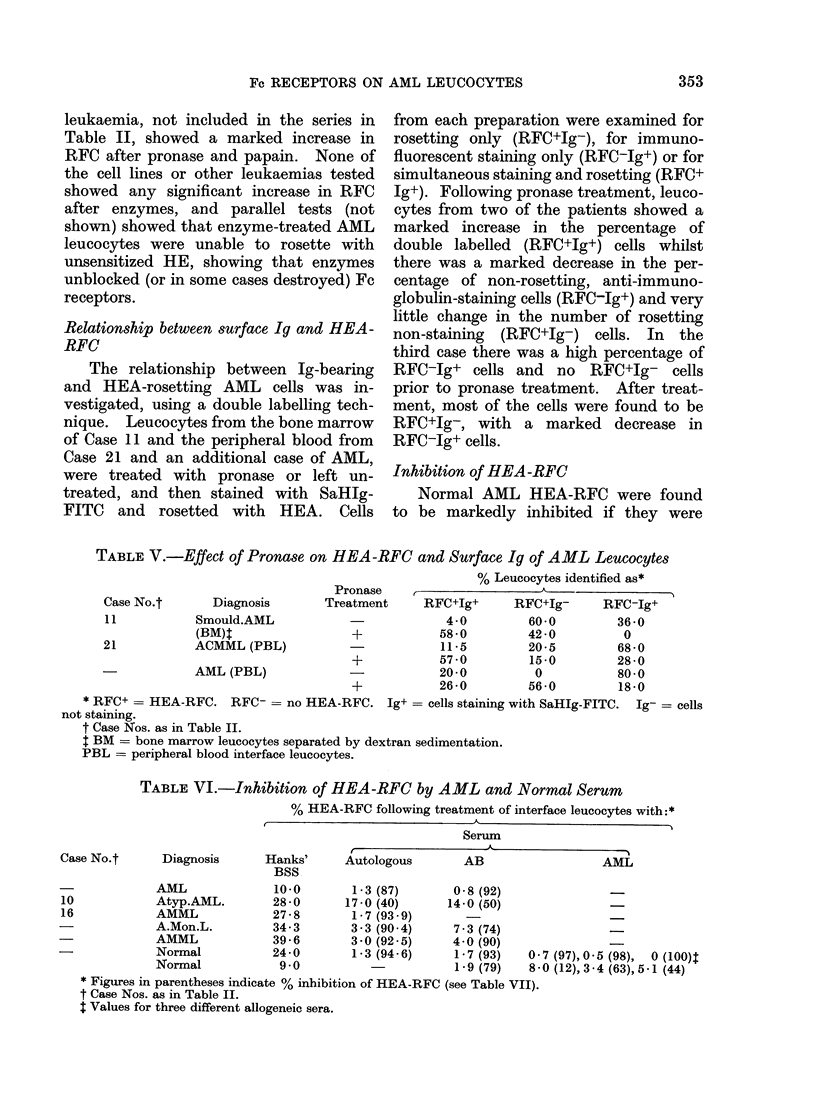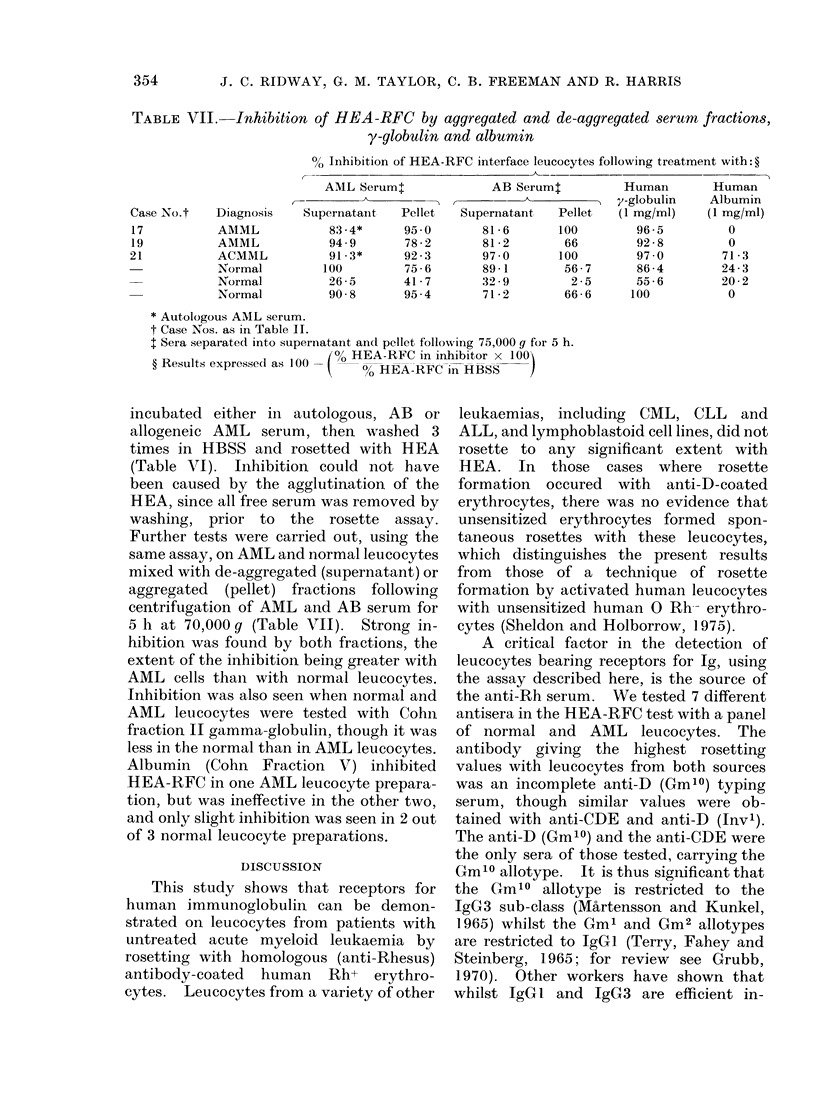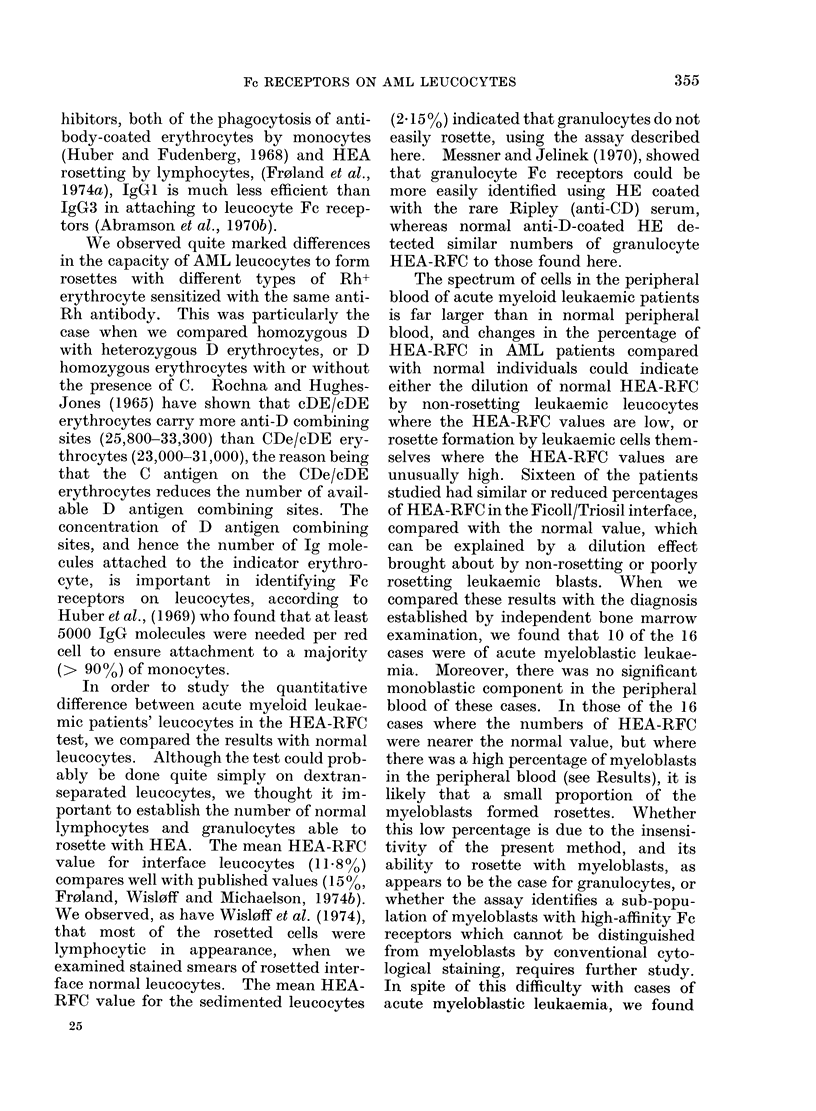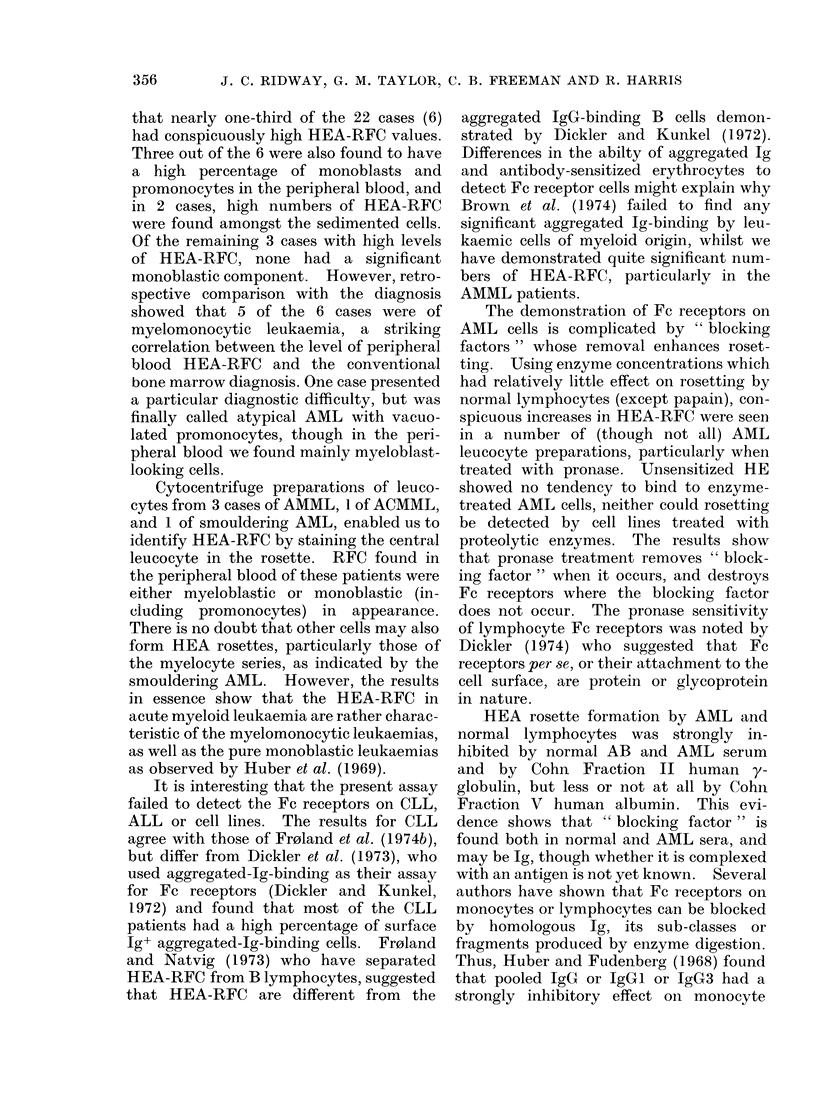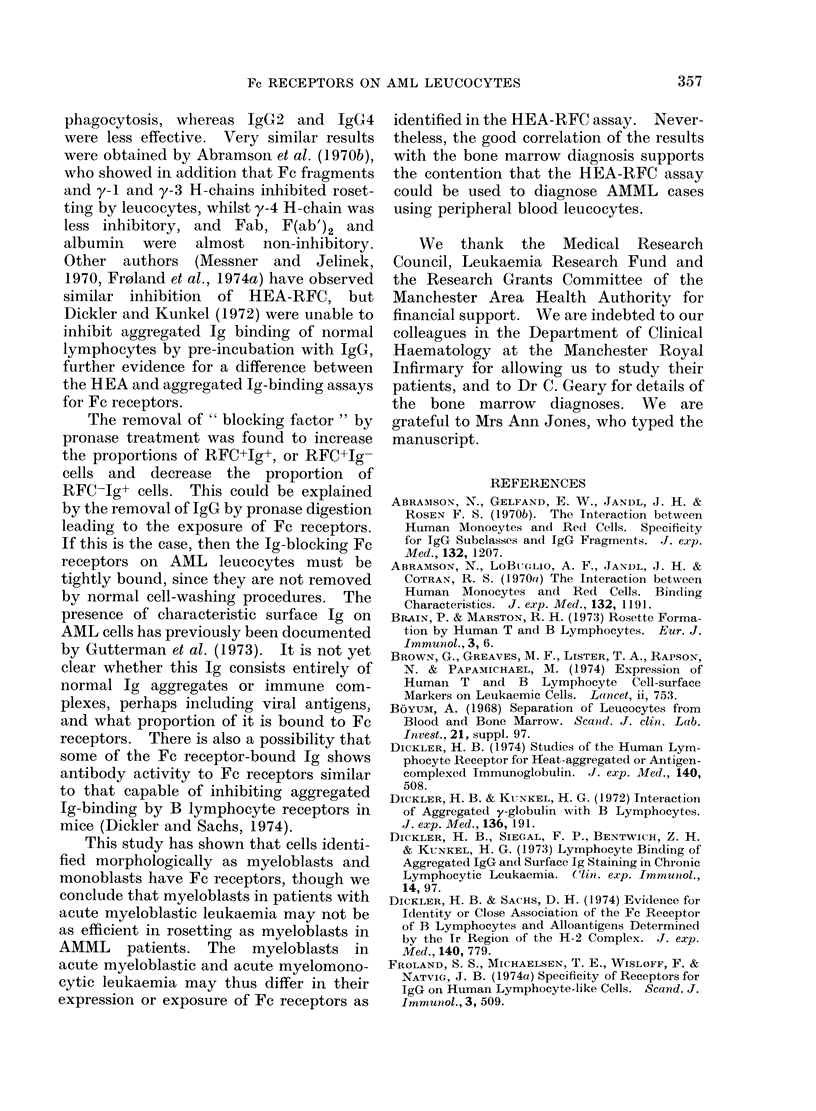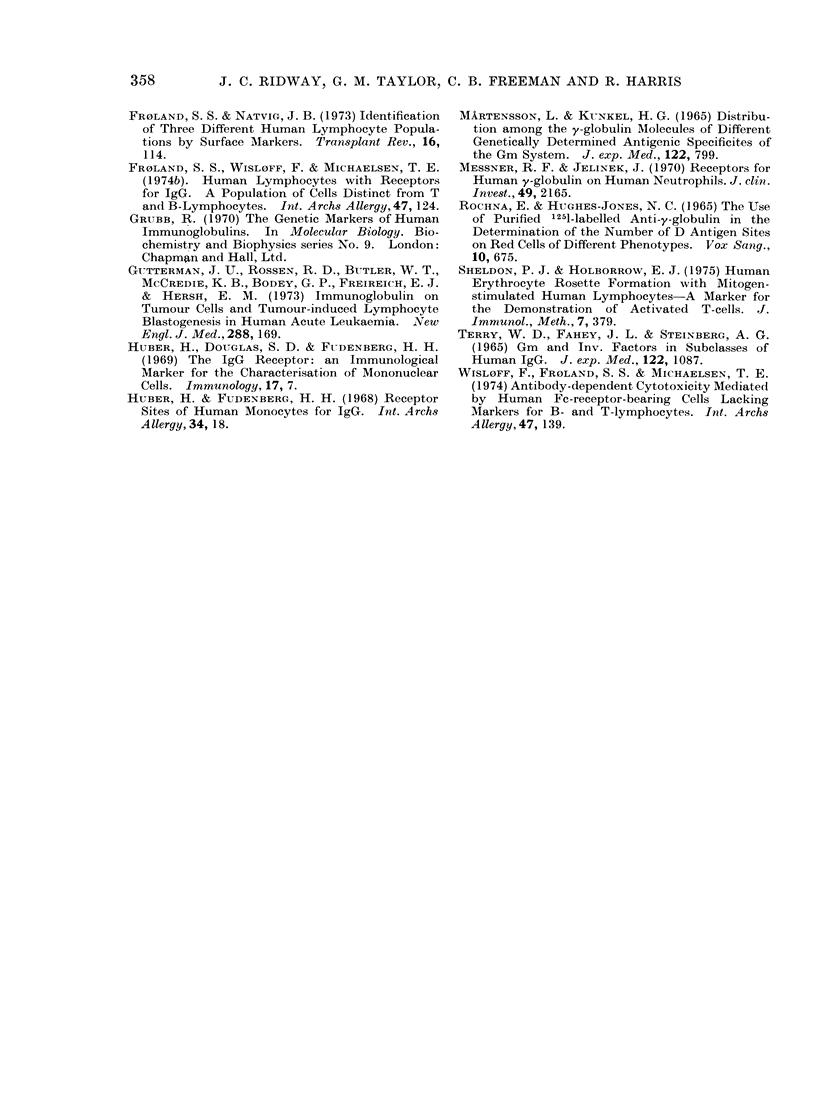# Receptors for human immunoglobulin on acute myeloid leukaemic leucocytes.

**DOI:** 10.1038/bjc.1976.177

**Published:** 1976-10

**Authors:** J. C. Ridway, G. M. Taylor, C. B. Freeman, R. Harris

## Abstract

Immunoglobulin (Fc) receptors were detected on leucocytes from patients with acute myeloid leukaemia (AML) by rosette formation with human cDE/cDE erthyrocytes (HE) sensitized with Rhesus (Rh) antisera (HEA). Of 7 Rh antisera tested, erythrocytes sensitized with anti-d (Gm10) detected the highest numbers of rosette-forming cells (HEA-RFC) in normal and AML leucocyte preparations. Using this assay, HEA-RFC was studied in 22 untreated AML patients and ce assay detected 11-6% lymphocyte and 2-1% granulocyte HEA-RFC in normal peripheral blood. Leucocytes from 16 to 22 AML patients had a similar or lower percentage than normal lymphocyte HEA-RFC, which could be explained by the dilution of peripheral blood leucocytes by poorly or non-rosetting leukaemic blasts. Ten of these 16 patients were diagnosed as having acute myeloblasts leukaemia. Six of the 22 AML patients had high HEA-RFC values of which 5 were diagnosed as having myelomonocytic leukaemia. Cytocentrifuge preparations of HEA-RFC showed that the proportion able to form rosettes was lower in myeloblasts than in monoblasts. Enzyme treatment (pronase), inhibition or simultaneous labelling of surface Ig and Fc receptors showed that the characteristic surface Ig found to AML cells is, at least in part, bound to Fc receptors. The HEA-RFC test described in this paper could be useful in the immuno-diagnosis of myelomonocytic leukaemia.


*Br. J. Cancer* (1976) **34**, 346

### RECEPTORS FOR HUMAN IMMUNOGLOBULIN ON ACUTE MYELOID LEUKAEMIC LEUCOCYTES

J. C. RIDWAY, G. M. TAYLOR, C. B. FREEMAN AND R. HARRIS

*From the University of Manchester, Department of Medical Genetics, St Mary's Hospital, Manchester, M13 0JH*

Received 9 March 1976 Accepted 8 June 1976

**Summary.—Immunoglobulin (Fc) receptors were detected on leucocytes from patients with acute myeloid leukaemia (AML) by rosette formation with human cDE/cDE erthyrocytes (HE) sensitized with Rhesus (Rh) antisera (HEA). Of 7 Rh antisera tested, erythrocytes sensitized with anti-D ($Gm^{10}$) detected the highest numbers of rosette-forming cells (HEA-RFC) in normal and AML leucocyte preparations. Using this assay, HEA-RFC was studied in 22 untreated AML patients and compared with results from 11 normal individuals, and other leukaemias. The assay detected 11·6% lymphocyte and 2·1% granulocyte HEA-RFC in normal peripheral blood. Leucocytes from 16 of the 22 AML patients had a similar or lower percentage than normal lymphocyte HEA-RFC, which could be explained by the dilution of peripheral blood leucocytes by poorly or non-rosetting leukaemic blasts. Ten of these 16 patients were diagnosed as having acute myeloblastic leukaemia. Six of the 22 AML patients had high HEA-RFC values of which 5 were diagnosed as having myelomonocytic leukaemia. Cytocentrifuge preparations of HEA-RFC showed that the proportion able to form rosettes was lower in myeloblasts than in monoblasts. Enzyme treatment (pronase), inhibition or simultaneous labelling of surface Ig and Fc receptors showed that the characteristic surface Ig found on AML cells is, at least in part, bound to Fc receptors. The HEA-RFC test described in this paper could be useful in the immuno-diagnosis of myelomonocytic leukaemia.**

Receptors for human IgG have been demonstrated on human leucocytes including monocytes (Huber and Fudenberg, 1968, Abramson *et al.*, 1970*a*), polymorphonuclear neutrophils (Messner and Jelinek, 1970) and a subpopulation of lymphocytes (Brain and Marston, 1973; Frøland *et al.*, 1974*a*). That the receptor recognizes the Fc fragment of IgG has been demonstrated by the inhibition of IgG binding to cells by isolated Fc but not Fab or F(ab')2 fragments (Abramson *et al.*, 1970*b*; Messner and Jelinek, 1970; Frøland *et al.*, 1974*a*). Although Fc receptors are important for the phagocytosis of antigen–antibody complexes by monocytes and macrophages, (Huber and Fudenberg, 1968, Huber, Douglas and Fudenberg, 1969) and in antibody-dependent lymphocyte-mediated cytotoxicity (Dickler, 1974; Wisløff, Frøland and Michaelson, 1974) the functional significance of Fc receptors *in vivo* still remains unclear. Nevertheless, Fc receptors are to be found on many cell types, both phagocytic and non-phagocytic, involved in the immune response.

We have observed, as have Gutterman *et al.* (1973) that cells from patients with acute myeloid leukaemia (AML)* often have a characteristic staining pattern when reacted with fluorescein-conjugated anti-human Ig. This irregular and patchy staining is similar to that seen when aggregated IgG bound to lymphocyte Fc receptors is stained with an anti-immunoglobulin fluorescent conjugate (Dickler

Address for Correspondence: Dr G. M. Taylor, Department of Medical Genetics, St Mary's Hospital, Hathersage Road, Manchester, M13 0JH.

* Unless specifically stated, AML is a generic term used in this paper—including all the acute myeloid leukaemias.

and Kunkel, 1972; Dickler, 1974). Like lymphocyte-bound aggregated IgG, but unlike lymphocyte surface Ig, AML-Ig can be capped only with difficulty or not at all (unpublished observations). This apparent similarity in the appearance and behaviour of lymphocyte-bound and AML-bound Ig led us to investigate whether AML cells also have Fc receptors. We used as indicator cells for Fc receptors, anti-Rhesus antibody-coated human erythrocytes. The results show that a number of patients, diagnosed on the basis of bone marrow cytology as acute myelomonocytic leukaemia (AMML) have large numbers of Fc receptor-bearing cells in the peripheral blood. Proteolytic enzyme or serum treatment of the AML cells show that these receptors can be further unmasked or blocked respectively, suggesting that at least part of the AML surface Ig is bound *in vivo* to Fc receptors. The results in this paper also show that the relatively rapid rosette test described could be useful in the diagnosis of AMML using peripheral blood leucocytes, as an adjunct to conventional bone marrow diagnosis.

### MATERIALS AND METHODS

*Normal and AML leucocyte separations.*—Venous blood from healthy adults and from untreated adult AML patients was anticoagulated with heparin (10 iu/ml) or by defibrination. Whole blood total white cell counts were performed on a Coulter Model " S " cell counter and differential cell counts were made using Romanowsky-stained blood smears. The blood was diluted 1 : 1 with saline and layered on to a mixture of Ficoll (Pharmacia, Uppsala, Sweden) and Triosil (Nyegaard, Oslo, Norway), centrifuged at 400 *g* for 40 min at 4° C (Böyum, 1968) and the leucocyte-enriched interface between the Ficoll/Triosil and plasma collected. The cells in this layer were then washed three times by centrifugation in Hanks' balanced salt solution (HBSS) before further use. The Ficoll/Triosil sedimented leucocytes were collected by resuspending the Ficoll/Triosil sedimented mixture of erythrocytes and leucocytes in 2 ml of supernatant plasma and 0·4 ml of 4·5% Dextran (Pharmacia, Uppsala, Sweden). The erythrocytes were sedimented at 4°C for approximately 30 min at 1 *g*, after which the leucocytes in the supernatant were removed and washed three times by centrifugation in HBSS. Smears of the cell separations were made on slides, stained with Romanowsky stain and differential cell counts made.

*Other leukaemias and cell lines.*—Peripheral blood leucocytes were obtained from chronic lymphocytic (CLL) acute lymphoblastic (ALL) and chronic myeloid (CML) leukaemic patients with high leucocyte counts. The leucocytes were separated on Ficoll/Triosil as above.

The two lymphoblastoid cell lines (BR18 and MICH) were obtained from Searle Laboratories, High Wycombe, and cultured in RPMI-1640 (Flow Laboratories, Scotland) containing 10% FCS.

*Human erythrocytes and Rh antisera.*—Human cDE/cDE ($R_2R_2$) erythrocytes (HE) from a single donor were collected into acid-citrate-dextrose solution and stored at 4°C for no longer than 1 month. Sera containing Rh antibody (Biotest, Folex, Birmingham, U.K.) in some cases of known Gm allotype were titrated against the HE, using the direct (saline) and indirect (Coombs) agglutination techniques. Of the 7 sera used, 2 (anti-D and anti-CDE) had complete and incomplete antibodies, whilst the other 5 had incomplete antibodies only. The highest dilution of Rh antiserum which gave the strongest indirect agglutination and no direct agglutination was selected for each serum as follows: anti-D, anti-CD, anti-CDE, all at 1/32; anti-D ($Gm^1$) 1/100; anti-D ($Gm^2$) 1/200; anti-D ($Gm^{10}$) and anti-D ($Inv^1$), both at 1/400.

*Sensitization of erythrocytes.*—Sensitization of HE for rosette assays and Gm typing was carried out by mixing equal volumes of appropriately diluted Rh antiserum with a 20% suspension of cDE/cDE erythrocytes at 37°C for 30 min, followed by three washes in phosphate-buffered saline (PBS). The antibody-coated erythrocytes (HEA) were resuspended in PBS at a concentration of $5 \times 10^7$/ml.

*Rosette-forming cell (RFC) assay.*—Aliquots ($3 \times 10^6$/ml) of the interface or sedimented cell suspensions were mixed with an equal volume of HEA in 13 × 76 mm plastic tubes. The mixtures were incubated at 20°C for 30 min, centrifuged at 200 *g* for 5 min and resuspended by gently tapping the tubes. Two drops of 0·2% methylene blue in saline were added to each cell suspension to stain the nucleated cells, and a single drop of this suspension was placed on a microscope slide. Rosetted cells were counted under bright field illumination, and the numbers expressed as a percentage of the total number of nucleated cells counted (300 +). A rosette was defined as a leucocyte surrounded by three or more HEA. In some cases cyto-centrifuge preparations of rosetted cells were made and stained with Romanowsky stain.

*Enzyme treatment of leucocytes.*—Equal volumes (0·1 ml) of leucocytes ($6 \times 10^6$/ml) and each of the following enzyme preparations in HBSS were incubated at 37°C for the following times: pronase (protease type VI, Sigma, London), 1 mg/ml—10 min; papain (Sigma, London), 1 mg/ml—10 min and trypsin (Wellcome Laboratories, Beckenham, Kent), 0·25 mg/ml—5 min. The enzyme-treated leucocytes were then washed three times in HBSS, resuspended at a concentration of $3 \times 10^6$/ml and rosetted with HEA.

*HEA-rosette inhibition.*—Leucocytes ($6 \times 10^6$/ml) from the interface of separated normal or AML blood were incubated for 30 min at 37°C with an equal volume (0·1 ml) of the following: normal AB or pretreatment AML serum centrifuged at 70,000 *g* for 5 h and separated into supernatant (deaggregated) and pellet fractions; human gamma-globulin (Cohn Fraction II, Sigma) or human albumin (Cohn Fraction V, Sigma), the latter two at concentrations of 1 mg/ml. The leucocytes were then washed by centrifugation three times in HBSS, resuspended at $3 \times 10^6$/ml and rosetted with HEA as previously described.

*Membrane immunofluorescence.*—AML leucocytes, with or without prior pronase treatment, were incubated with 1/10 dilution of sheep anti-human immunoglobulin-FITC conjugate (SaHIg-FITC, Wellcome Laboratories, Beckenham, Kent) at 4°C for 30 min. The leucocytes were washed three times with HBSS and rosetted with HEA, suspended in PBS, and each cell examined under alternate dark field u.v. light for fluorescing cells and bright field phase contrast for rosetted cells. These cells were classified either as rosetting but not staining (RFC + Ig—), non-rosetting but staining (RFC — Ig +, or those both rosetting and staining (RFC + Ig +).

### RESULTS

### *Optimum HEA-RFC technique*

The optimum method for HEA rosette formation with AML and normal leucocytes, was found to be 30 min incubation at 20°C, followed by centrifugation at 200 *g* for 5 min. Centrifuging cells prior to incubation did not affect the results, but incubation at 37°C gave lower values.

The source of the Rh antisera used to sensitize the cDE/cDE erythrocytes, was found to be particularly important. In Table 1 results are shown for leucocytes from 5 normal individuals and 2 AMML patients when tested for HEA with erythrocytes coated with different anti-Rhesus antibodies.

Anti-D ($Gm^{10}$) sensitized cDE/cDE erythrocytes gave consistently higher values than the other sensitizing sera. Note also the higher RFC values obtained with the AMML (case 15) compared with normal leucocytes. Two anti-D ($Gm^{10}$) sera from different donors were used to sensitize different batches of HE, and were found to give comparable results (not shown). The Gm allotypes of the Rh antisera were determined by direct agglutination of HEA with anti-Gm typing

TABLE I.—*Rosette Formation by Normal and AML Leucocytes with HE sentisized with Different Anti-rhesus Sera*

| Peripheral blood leucocytes from: | Ficoll/Triosil fraction | % leucocytes forming rosettes with HE sensitized with:† | | | | | | |
|---|---|---|---|---|---|---|---|---|
| | | Anti-D | Anti-CD | Anti-CDE | Anti-D ($Gm^1$) | Anti-D ($Gm^2$) | Anti-D ($Gm^{10}$) | Anti-D ($Inv^1$) |
| Normal‡ | Interface | 3·3 | 2·8 | 5·3 | 3·9 | 3·2 | 13·0 | 10·8 |
| AMML (15)* | Interface | 8·8 | 5·6 | 38·7 | 8·7 | 9·6 | 43·2 | 34·5 |
| ?AMML (18)* | Interface | 3·3 | 6·2 | 7·2 | 1·0 | 1·7 | 11·1 | 5·2 |

* Case Nos. from Table II.

† All sera from Biotest Folex U.K.

‡ Average of tests on 5 normals.

TABLE II.—*Rosette Formation by Normal, AML, and Other Leucocytes with Anti-D* (*Gm*[10]) *HEA*

| | | Peripheral blood before separation | | | | | | | Ficoll/Triosil separation‡ | | | | | |
|---|---|---|---|---|---|---|---|---|---|---|---|---|---|---|
| | | | % Cells identified as:§ | | | | | | Interface | | | Sediment | | |
| Case No. | Diagnosis* | TWCC/mm³ × 10⁻³ | L | N | M | My | MyBl | MoBl/PMO | % RFC | % Bl | % Oth | % RFC | % Bl | % Oth |
| 1 | AML | 1·8 | 58 | 4 | 0 | 0 | 34 | 0 | 14·6 | — | — | — | — | — |
| 2 | AML | 1·9 | 82 | 14 | 0 | 0 | 2 | 0 | 1·0 | — | — | — | — | — |
| 3 | AML | 3·9 | 79 | 2 | 0 | 0 | 19 | 0 | 2·0 | 17 | 83 | — | — | — |
| 4 | AML | 24·7 | 17 | 2 | 5 | 12 | 55 | 0/9 | 8·9 | 83 | 17 | — | — | — |
| 5 | AML | 27·9 | 12 | 0 | 0 | 0 | 88 | 0 | 4·5 | — | — | 1·0 | — | — |
| 6 | AML | 136·9 | 6 | 2 | 0 | 2 | 90 | 0 | 10·0 | 82 | 18 | 5·0 | 42 | 48 |
| 7 | AML | 182·0 | 12 | 2 | 0 | 4 | 81 | 0 | 0·3 | 90 | 10 | 8·3 | — | — |
| 8 | Erythro.AML | 4·0 | 20 | 0 | 1 | 1 | 77 | 0 | 2·0 | — | — | 1·7 | — | — |
| 9 | Erythro.AML | 30·1 | 10 | 26 | 1 | 10 | 53 | 0 | 13·6 | 81 | 19 | — | — | — |
| 10 | Atyp.AML. | 20·3 | 27 | 3 | 0 | 0 | 63 | 0 | 28·0 | 66 | 34 | — | — | — |
| 11 | Smould.AML → AML | 24·8 | 5 | 52 | 0 | 10 | 36 | 0 | 15·0 | — | — | 16·0 | — | — |
| 12 | AMML | 2·7 | 26 | 0 | 2 | 12 | 60 | 0 | 0·0 | — | — | 0·3 | — | — |
| 13 | AMML | 2·8 | 45 | 18 | 6 | 5 | 18 | 0 | 0·0 | — | — | 1·2 | 0 | 100 |
| 14 | AMML | 12·6 | 26 | 13 | 0 | 12 | 48 | 0 | 8·0 | — | — | — | — | — |
| 15 | AMML | 17·2 | 35 | 5 | 5 | 3 | 50 | 0/2 | 43·2 | 75 | 25 | 12·9 | 2 | 98 |
| 16 | AMML | 19·7 | 17 | 3 | 4 | 17 | 10 | 49 | 27·8 | 77 | 27 | 32·3 | — | — |
| 17 | ?AMML | 25·8 | 9 | 5 | 6 | 0 | 2 | 35/43 | 63·7 | 87 | 13 | 68·2 | 0 | 100 |
| 18 | ?AMML | 28·9 | 19 | 6 | 2 | 3 | 70 | 0 | 11·1 | 86 | 14 | 3·7 | 8 | 92 |
| 19 | AMML | 41·7 | 4 | 5 | 0 | 8 | 83 | 0 | 59·2 | 84 | 16 | 62·7 | — | — |
| 20 | AMML | 482·6 | 2 | 1 | 0 | 0 | 85 | 0/10 | 1·4 | 100 | 0 | 5·2 | 35 | 65 |
| 21 | ACMML | 136·0 | 5 | 12 | 12 | 11 | 15 | 40/0 | 50·0 | 80 | 20 | 7·0 | 0 | 100 |
| 22 | Atyp. MonoL. | 34·6 | 34 | 14 | 52 | 0 | 0 | 0 | 2·7 | 0 | 100 | — | — | — |
| 23 | CLL | 198·7 | | | | | | | 0·0 | | | | | |
| 24 | CLL | 272·2 | | | | | | | 0·0 | | | | | |
| 25 | AL/Adult | 46·2 | | | | | | | 0·3 | | | | | |
| 26 | CML | 157·0 | 1 | 63 | 0 | 28 | 3 | 0 | 2·6 | | | | | |
| 27 | CML | 580·0 | 2 | 49 | 1 | 39 | 1 | 0 | 6·2 | | | | | |
| 28 | CML | 362·0 | 4 | 39 | 1 | 47 | 6 | 0 | 9·2 | | | | | |
| 29 | CML | 700·0 | 1 | 35 | 0 | 60 | 1 | 0 | 4·3 | | | | | |
| | | Normal (n = 11)† | | | | | | | 11·64 ±4·24 | — | L§ 84 ±14 | 2·15 ±1·22 | — | N§ 95·8 ±7 |

Abbreviations: * AML, acute myeloblastic leukaemia; Erythro.AML, erythroid AML; Atyp.AML, atypical AML; Atyp.MonoL., atypical monoblastic leukaemia; Smould. AML, smouldering AML; AMML, acute myelomonocytic leukaemia; ACMML, acute on chronic myelomonocytic leukaemia; CLL, chronic lymphocytic leukaemia; ALL, acute lymphoblastic leukaemia; CML, chronic myeloid leukaemia.

† Mean values ± s.d.

‡% Bl, percentage myeloblasts, monoblasts and promonocytes; %Oth, percentage others including L, N, M, and myeloid precursors exclusive of blasts.

§ L, lymphocytes; N, neutrophils; M, monocytes; My, includes promyelocytes, myelocytes and metamyelocytes; MyBl, myeloblasts; MoBl/PMO, Monoblasts/Promonocytes.

TWCC total white cell counts.

sera. Only the anti-D ($Gm^{10}$) and the anti-CDE sera reacted with anti-$Gm^{10}$ serum. The significance of this reaction is discussed later.

In further tests (data not shown), HE of different Rhesus phenotypes were sensitized with Anti-D ($Gm^{10}$) antiserum and tested with 4 different AMML cells. The percentage RFC obtained was lower when d/D rather than D/D indicator cells were used in the RFC assay. Moreover, the presence of the C phenotype reduced the number of RFC. Maximum numbers of RFC were found with cDE/cDE erythrocytes, and no RFC were detected using unsensitized cDE/cDE erythrocytes, or anti-D ($Gm^{10}$) treated cde/cde erythrocytes.

*Rosette formation by normal and AML leucocytes*

Using anti-D ($Gm^{10}$) sensitized cDE/cDE erythrocytes as indicator cells, receptors for human Ig were investigated on leucocytes from 11 normal individuals, 22 with untreated acute myeloid leukaemia, 2 with chronic lymphocytic leukaemia, 4 with chronic myeloid leukaemia, and one with adult lymphoblastic leukaemia. The results are depicted in Table II. The diagnoses, which in the case of the myeloid leukaemias were established by the examination of Romanowsky-stained bone marrow smears in the Department of Clinical Haematology, at the Manchester Royal Infirmary, were in most cases unknown to us at the time that peripheral blood leucocytes were assayed for HEA-RFC. The differential cell counts were performed in our own laboratory on the blood samples subsequently separated on Ficoll/Triosil, and in a number of cases differential counts were performed on the cells obtained after Ficoll/Triosil separation.

Blasts from the AML cell separations were found to accumulate mainly in the interface fraction, unless the peripheral leucocyte count was greater than $5 \times 10^4/mm^3$, in which case they were carried over into the sedimented fraction. In the majority of cases examined after Ficoll/Triosil separation, there was an increase in the proportion of blasts in the interface fraction compared with the unseparated cells. In the 5 preparations of sedimented cells examined, nucleated cells other than blasts comprised the majority of cells.

Ficoll/Triosil separation is a method principally for separating lymphocytes from polymorphonuclear neutrophils (also referred to in this paper as "granulocytes") and erythrocytes. To define the number of HEA-RFC in these two leucocyte fractions, results of 11 separations of normal leucocytes are shown. Nine of the 11 interface separations contained 80% or more (mean 84%) lymphocytes, most of the other cells being granulocytes, whilst all 11 Ficoll/Triosil-sedimented leucocytes contained 75% or more (mean 95·8%) granulocytes, both fractions being assessed by differential counting of Romanowsky-stained smears.

The normal leucocyte fractions were rosetted to establish an HEA-RFC baseline with which to compare AML HEA-RFC values. If greater percentages of RFC are found in AML peripheral leucocyte preparations, compared with normal leucocytes, this could be taken to indicate the participation of AML cells in rosette formation, whilst any decrease in AML RFC compared with normal could mean the dilution of normal RFC by non-rosetting leukaemic leucocytes. In agreement with Table I, interface normal leucocytes had greater numbers of RFC ($11{\cdot}64 \pm 4{\cdot}24\%$) than the sedimented granulocytes ($2{\cdot}15 \pm 1{\cdot}22\%$) separated from the same blood sample (Table II). Sixteen of the 22 cases of AML had RFC values within or lower than the normal interface range. In most cases where there was a high percentage of myeloblasts and a very low number of RFC (Cases 5, 7, 8, 12 and 20) myeloblasts were not likely on numerical grounds to be forming rosettes. In other cases, (1, 4, 6, 9, 11, 14 and 18) where the RFC values approximate to the "normal", the unseparated and separated cells contained relatively high percentages of myeloblasts. This could mean, either that all or most of the lymphocytes present in these patients were of the minor sub-population found in normals bearing Fc receptors, or more likely that a small number of other nucleated cells, including myeloblasts were HEA-RFC. It is not possible to distinguish between these two possibilities. Nevertheless, 8 of the 12 cases with relatively low numbers of RFC, but with relatively high numbers of blasts described above, were diagnosed as acute myeloblastic leukaemia.

Six AML cases had conspicuously high numbers of RFC (cases 10, 15, 16, 17, 19 and 21) of which 3 (15, 16 and 19) were confirmed, and 1 (17) was suspected AMML by bone marrow examination. Case 21 was one of chronic myelomonocytic leukaemia in blast crisis. Two cases (15 and 19) had no significant monoblastic, monocytic or promonocytic component in the peripheral blood assessed by cytological examination, whereas cases 16, 17 and 21 had a high proportion of mixed monoblasts and promonocytes and fewer myeloblasts. Further details of cases 17, 19 and 21 are presented below. Case 10, which had abnormal myeloblasts in the bone marrow, was diagnosed as a " vacuolated promonocytic " AML.

Fourteen sedimented AML leucocyte fractions were tested and 3 (16, 17 and 19) had high numbers of HEA-RFC similar to those in the interface of the same preparation. In the remaining cases, the HEA-RFC values were similar to or lower than those found in the interface fraction. One case tested, of atypical monocytic leukaemia (22), did not have the large numbers of HEA-RFC which might have been expected from the diagnosis and the peripheral blood differential count.

The CLLs, CMLs and the ALL all had HEA-RFC values lower than "normal", although in real terms the numbers of HEA-RFC were high in CML peripheral blood.

*Cytology of HEA-RFC*

Rosetted AML leucocytes were identified in Romanowsky-stained cytocentrifuge preparations. Many of the preparations proved equivocal, because of the tendency of rosetted leucocytes to condense and " overstain ", and the presence of false rosettes caused by clustering of HE around leucocytes during drying. However, 5 preparations were made where these problems were not encountered, and the results are shown in Table III.

The smouldering leukaemia (Case No. 11) had predominantly myelocyte HEA-RFC in the interface fraction, though 10% of the RFC were myeloblasts. The sedimented cells had predominantly granulocyte RFC, with 30% myelocyte RFC, but no myeloblast RFC. Cases 17 and 18, both diagnosed by bone marrow examination, as probable AMML, differed in the numbers of HEA-RFC in the peripheral blood (see Table II). In case 17, with 63% HEA-RFC in the interface and 68% HEA-RFC in the sediment, most of the rosetted cells were monocytic in appearance (Table III), though 40% of the sedimented RFC were granulocytes. No myeloblast RFC were seen in this preparation, which is not

TABLE III.—*Cytology of Rosetted AML Leucocytes*

| Case No. | Diagnosis* | Ficoll/Triosil fraction | % HEA-RFC identified as:* L | N | M + MoBl | My | MyBl | Unident |
|---|---|---|---|---|---|---|---|---|
| 11 | Smould. AML | Interface | 0 | 5 | 0 | 55 | 10 | 30 |
| | | Sediment | 0 | 70 | 0 | 30 | 0 | 0 |
| 17 | ?AMML | Interface | 0 | 0 | 70 | 0 | 0 | 30 |
| | | Sediment | 0 | 40 | 60 | 0 | 0 | 0 |
| 18 | ?AMML | Interface | 0 | 0 | 0 | 0 | 100 | 0 |
| 19 | AMML | Interface | 6 | 0 | 10 | 0 | 83 | 1 |
| 21 | ACMML | Interface | 0 | 0 | 62 | 0 | 38 | 0 |

* Abbreviations and case numbers as in Table II. Unident: unidentifiable.

surprising, since myeloblasts were only identified as 20% of the peripheral blood leucocytes in the unseparated sample. Case 18 was quite different, since monocytic cells were a very small percentage, and myeloblasts a high percentage, of the unseparated peripheral blood leucocytes. The HEA-RFC in the interface (11·1%; Table II) were all myeloblasts (Table III). Since 86% of the interface cells were myeloblasts, of which 11% were rosetted, about 70% of the myeloblasts did not form rosettes using the HEA-RFC assay. Case 19, a confirmed AMML, had 59·2% HEA-RFC (Table II) most of which proved to be myeloblasts in the cytocentrifuge preparations (83%; Table III). Case 21, that of ACMML, had a mixture of nearly twice as many monocytic RFC as myeloblastic RFC.

*Effect of enzymes on HEA-RFC*

To investigate whether rosetting could be increased by the removal of Fc receptor-bound immunoglobulin, the effect of proteolytic enzymes on normal and AML leucocytes was compared in the following experiments. Preliminary tests had shown that concentrations of trypsin above 0·25 mg/ml reduced the HEA-RFC in normal leucocyte preparations. Normal and AML leucocytes were thus incubated for 5 min at 37°C in 0·25 mg/ml trypsin in HBSS, and in similar experiments for 10 min at 37°C in 1 mg/ml papain or 1 mg/ml pronase in HBSS. The results in Table IV show that interface leucocytes from normal individuals showed a slight decrease in RFC after proteolytic enzyme treatment, particularly papain, whilst normal sedimented granulocytes showed a small increase in RFC.

Interface leucocytes from 6/8 and sedimented leucocytes from 3/5 AML patients showed a marked increase in HEA-RFC following treatment with proteolytic enzymes, particularly after pronase and to a lesser extent papain. This increase was most marked in case 16, an AMML with a low percentage of myeloblasts, but a high percentage of monoblasts/promonocytes, and was much less apparent, or non-existent, in the enzyme-treated acute myeloblastic leukaemias. The case of ACMML (Case 21) showed a decrease in RFC numbers after pronase treatment, whilst one of acute monoblastic

TABLE IV.—*Effect of Proteolytic Enzymes on AML HEA-RFC*

| Case No.* | Diagnosis | Ficoll/Triosil fraction | % of HEA-RFC following treatment with: | | | |
|---|---|---|---|---|---|---|
| | | | HBSS | Pronase | Papain | Trypsin |
| 5 | AML | Interface | 4·5 | 7·6 | 10·7 | —‡ |
| | | Sediment | 1·0 | 7·6 | — | — |
| 6 | AML | Interface | 10·0 | 2·7 | 4·0 | 2·0 |
| | | Sediment | 5·0 | 2·7 | 2·0 | 0·3 |
| 8 | Erythro.AML | Interface | 2·0 | 2·0 | 2·3 | 1·7 |
| 11 | Smould.AML | Interface | 15·0 | 26·0 | — | 16·0 |
| | | Sediment | 16·0 | 29·6 | — | 23·7 |
| — | Smould.AML | Interface | 6·0 | 8·0 | 6·0 | 4·2 |
| 12 | AMML | Interface | 0 | 4·7 | 2·6 | 5·3 |
| 16 | AMML | Interface | 27·8 | 50·3 | 20·7 | — |
| 21 | ACMML | Interface | 50·0 | 39·8 | — | — |
| | | Sediment | 7·0 | 4·3 | — | — |
| — | A.Mono.L. | Interface | 2·7 | 17·3 | 14·5 | 5·3 |
| — | CLL | | 0 | 0 | 0 | 0 |
| — | ALL | | 0·3 | 0·3 | 0·6 | 0·6 |
| — | BRI8 } § | | 0 | 0 | 0 | 0 |
| — | MICH } § | | | | | |
| | Normal† | Interface | 13·1±5·9 | 9·2±7·4 | 4·6±3·6 | 8·0±6·3 |
| | (n = 7) | Sediment | 2·9±2·2 | 5·2±4·2 | 2·5±2·1 | 3·1±3·1 |

* Case nos. as Table II.
† Mean values ± S.D.
‡ — not tested.
§ Lymphoblastoid cell lines.

leukaemia, not included in the series in Table II, showed a marked increase in RFC after pronase and papain. None of the cell lines or other leukaemias tested showed any significant increase in RFC after enzymes, and parallel tests (not shown) showed that enzyme-treated AML leucocytes were unable to rosette with unsensitized HE, showing that enzymes unblocked (or in some cases destroyed) Fc receptors.

*Relationship between surface Ig and HEA-RFC*

The relationship between Ig-bearing and HEA-rosetting AML cells was investigated, using a double labelling technique. Leucocytes from the bone marrow of Case 11 and the peripheral blood from Case 21 and an additional case of AML, were treated with pronase or left untreated, and then stained with SaHIg-FITC and rosetted with HEA. Cells from each preparation were examined for rosetting only ($RFC^+Ig^-$), for immunofluorescent staining only ($RFC^-Ig^+$) or for simultaneous staining and rosetting ($RFC^+Ig^+$). Following pronase treatment, leucocytes from two of the patients showed a marked increase in the percentage of double labelled ($RFC^+Ig^+$) cells whilst there was a marked decrease in the percentage of non-rosetting, anti-immunoglobulin-staining cells ($RFC^-Ig^+$) and very little change in the number of rosetting non-staining ($RFC^+Ig^-$) cells. In the third case there was a high percentage of $RFC^-Ig^+$ cells and no $RFC^+Ig^-$ cells prior to pronase treatment. After treatment, most of the cells were found to be $RFC^+Ig^-$, with a marked decrease in $RFC^-Ig^+$ cells.

*Inhibition of HEA-RFC*

Normal AML HEA-RFC were found to be markedly inhibited if they were

TABLE V.—*Effect of Pronase on HEA-RFC and Surface Ig of AML Leucocytes*

| Case No.† | Diagnosis | Pronase Treatment | % Leucocytes identified as* $RFC^+Ig^+$ | $RFC^+Ig^-$ | $RFC^-Ig^+$ |
|---|---|---|---|---|---|
| 11 | Smould.AML | — | 4·0 | 60·0 | 36·0 |
| | (BM)‡ | + | 58·0 | 42·0 | 0 |
| 21 | ACMML (PBL) | — | 11·5 | 20·5 | 68·0 |
| | | + | 57·0 | 15·0 | 28·0 |
| — | AML (PBL) | — | 20·0 | 0 | 80·0 |
| | | + | 26·0 | 56·0 | 18·0 |

* $RFC^+$ = HEA-RFC. $RFC^-$ = no HEA-RFC. $Ig^+$ = cells staining with SaHIg-FITC. $Ig^-$ = cells not staining.
† Case Nos. as in Table II.
‡ BM = bone marrow leucocytes separated by dextran sedimentation.
PBL = peripheral blood interface leucocytes.

TABLE VI.—*Inhibition of HEA-RFC by AML and Normal Serum*

| Case No.† | Diagnosis | % HEA-RFC following treatment of interface leucocytes with:* Hanks' BSS | Serum: Autologous | AB | AML |
|---|---|---|---|---|---|
| — | AML | 10·0 | 1·3 (87) | 0·8 (92) | — |
| 10 | Atyp.AML. | 28·0 | 17·0 (40) | 14·0 (50) | — |
| 16 | AMML | 27·8 | 1·7 (93·9) | — | — |
| — | A.Mon.L. | 34·3 | 3·3 (90·4) | 7·3 (74) | — |
| — | AMML | 39·6 | 3·0 (92·5) | 4·0 (90) | — |
| — | Normal | 24·0 | 1·3 (94·6) | 1·7 (93) | 0·7 (97), 0·5 (98), 0 (100)‡ |
| | Normal | 9·0 | — | 1·9 (79) | 8·0 (12), 3·4 (63), 5·1 (44) |

* Figures in parentheses indicate % inhibition of HEA-RFC (see Table VII).
† Case Nos. as in Table II.
‡ Values for three different allogeneic sera.

TABLE VII.—*Inhibition of HEA-RFC by aggregated and de-aggregated serum fractions, γ-globulin and albumin*

| | | % Inhibition of HEA-RFC interface leucocytes following treatment with:§ | | | | | |
|---|---|---|---|---|---|---|---|
| | | AML Serum‡ | | AB Serum‡ | | Human γ-globulin (1 mg/ml) | Human Albumin (1 mg/ml) |
| Case No.† | Diagnosis | Supernatant | Pellet | Supernatant | Pellet | | |
| 17 | AMML | 83·4* | 95·0 | 81·6 | 100 | 96·5 | 0 |
| 19 | AMML | 94·9 | 78·2 | 81·2 | 66 | 92·8 | 0 |
| 21 | ACMML | 91·3* | 92·3 | 97·0 | 100 | 97·0 | 71·3 |
| — | Normal | 100 | 75·6 | 89·1 | 56·7 | 86·4 | 24·3 |
| — | Normal | 26·5 | 41·7 | 32·9 | 2·5 | 55·6 | 20·2 |
| — | Normal | 90·8 | 95·4 | 71·2 | 66·6 | 100 | 0 |

* Autologous AML serum.
† Case Nos. as in Table II.
‡ Sera separated into supernatant and pellet following 75,000 $g$ for 5 h.
§ Results expressed as $100 - \left(\frac{\% \text{ HEA-RFC in inhibitor} \times 100}{\% \text{ HEA-RFC in HBSS}}\right)$

incubated either in autologous, AB or allogeneic AML serum, then washed 3 times in HBSS and rosetted with HEA (Table VI). Inhibition could not have been caused by the agglutination of the HEA, since all free serum was removed by washing, prior to the rosette assay. Further tests were carried out, using the same assay, on AML and normal leucocytes mixed with de-aggregated (supernatant) or aggregated (pellet) fractions following centrifugation of AML and AB serum for 5 h at 70,000 $g$ (Table VII). Strong inhibition was found by both fractions, the extent of the inhibition being greater with AML cells than with normal leucocytes. Inhibition was also seen when normal and AML leucocytes were tested with Cohn fraction II gamma-globulin, though it was less in the normal than in AML leucocytes. Albumin (Cohn Fraction V) inhibited HEA-RFC in one AML leucocyte preparation, but was ineffective in the other two, and only slight inhibition was seen in 2 out of 3 normal leucocyte preparations.

### DISCUSSION

This study shows that receptors for human immunoglobulin can be demonstrated on leucocytes from patients with untreated acute myeloid leukaemia by rosetting with homologous (anti-Rhesus) antibody-coated human $Rh^+$ erythrocytes. Leucocytes from a variety of other leukaemias, including CML, CLL and ALL, and lymphoblastoid cell lines, did not rosette to any significant extent with HEA. In those cases where rosette formation occured with anti-D-coated erythrocytes, there was no evidence that unsensitized erythrocytes formed spontaneous rosettes with these leucocytes, which distinguishes the present results from those of a technique of rosette formation by activated human leucocytes with unsensitized human O $Rh^-$ erythrocytes (Sheldon and Holborrow, 1975).

A critical factor in the detection of leucocytes bearing receptors for Ig, using the assay described here, is the source of the anti-Rh serum. We tested 7 different antisera in the HEA-RFC test with a panel of normal and AML leucocytes. The antibody giving the highest rosetting values with leucocytes from both sources was an incomplete anti-D ($Gm^{10}$) typing serum, though similar values were obtained with anti-CDE and anti-D ($Inv^1$). The anti-D ($Gm^{10}$) and the anti-CDE were the only sera of those tested, carrying the $Gm^{10}$ allotype. It is thus significant that the $Gm^{10}$ allotype is restricted to the IgG3 sub-class (Mårtensson and Kunkel, 1965) whilst the $Gm^1$ and $Gm^2$ allotypes are restricted to IgG1 (Terry, Fahey and Steinberg, 1965; for review see Grubb, 1970). Other workers have shown that whilst IgG1 and IgG3 are efficient inhibitors, both of the phagocytosis of antibody-coated erythrocytes by monocytes (Huber and Fudenberg, 1968) and HEA rosetting by lymphocytes, (Frøland *et al.*, 1974*a*), IgG1 is much less efficient than IgG3 in attaching to leucocyte Fc receptors (Abramson *et al.*, 1970*b*).

We observed quite marked differences in the capacity of AML leucocytes to form rosettes with different types of $Rh^+$ erythrocyte sensitized with the same anti-Rh antibody. This was particularly the case when we compared homozygous D with heterozygous D erythrocytes, or D homozygous erythrocytes with or without the presence of C. Rochna and Hughes-Jones (1965) have shown that cDE/cDE erythrocytes carry more anti-D combining sites (25,800–33,300) than CDe/cDE erythrocytes (23,000–31,000), the reason being that the C antigen on the CDe/cDE erythrocytes reduces the number of available D antigen combining sites. The concentration of D antigen combining sites, and hence the number of Ig molecules attached to the indicator erythrocyte, is important in identifying Fc receptors on leucocytes, according to Huber *et al.*, (1969) who found that at least 5000 IgG molecules were needed per red cell to ensure attachment to a majority ($> 90\%$) of monocytes.

In order to study the quantitative difference between acute myeloid leukaemic patients' leucocytes in the HEA-RFC test, we compared the results with normal leucocytes. Although the test could probably be done quite simply on dextran-separated leucocytes, we thought it important to establish the number of normal lymphocytes and granulocytes able to rosette with HEA. The mean HEA-RFC value for interface leucocytes (11·8%) compares well with published values (15%, Frøland, Wisløff and Michaelson, 1974*b*). We observed, as have Wisløff *et al.* (1974), that most of the rosetted cells were lymphocytic in appearance, when we examined stained smears of rosetted interface normal leucocytes. The mean HEA-RFC value for the sedimented leucocytes (2·15%) indicated that granulocytes do not easily rosette, using the assay described here. Messner and Jelinek (1970), showed that granulocyte Fc receptors could be more easily identified using HE coated with the rare Ripley (anti-CD) serum, whereas normal anti-D-coated HE detected similar numbers of granulocyte HEA-RFC to those found here.

The spectrum of cells in the peripheral blood of acute myeloid leukaemic patients is far larger than in normal peripheral blood, and changes in the percentage of HEA-RFC in AML patients compared with normal individuals could indicate either the dilution of normal HEA-RFC by non-rosetting leukaemic leucocytes where the HEA-RFC values are low, or rosette formation by leukaemic cells themselves where the HEA-RFC values are unusually high. Sixteen of the patients studied had similar or reduced percentages of HEA-RFC in the Ficoll/Triosil interface, compared with the normal value, which can be explained by a dilution effect brought about by non-rosetting or poorly rosetting leukaemic blasts. When we compared these results with the diagnosis established by independent bone marrow examination, we found that 10 of the 16 cases were of acute myeloblastic leukaemia. Moreover, there was no significant monoblastic component in the peripheral blood of these cases. In those of the 16 cases where the numbers of HEA-RFC were nearer the normal value, but where there was a high percentage of myeloblasts in the peripheral blood (see Results), it is likely that a small proportion of the myeloblasts formed rosettes. Whether this low percentage is due to the insensitivity of the present method, and its ability to rosette with myeloblasts, as appears to be the case for granulocytes, or whether the assay identifies a sub-population of myeloblasts with high-affinity Fc receptors which cannot be distinguished from myeloblasts by conventional cytological staining, requires further study. In spite of this difficulty with cases of acute myeloblastic leukaemia, we found that nearly one-third of the 22 cases (6) had conspicuously high HEA-RFC values. Three out of the 6 were also found to have a high percentage of monoblasts and promonocytes in the peripheral blood, and in 2 cases, high numbers of HEA-RFC were found amongst the sedimented cells. Of the remaining 3 cases with high levels of HEA-RFC, none had a significant monoblastic component. However, retrospective comparison with the diagnosis showed that 5 of the 6 cases were of myelomonocytic leukaemia, a striking correlation between the level of peripheral blood HEA-RFC and the conventional bone marrow diagnosis. One case presented a particular diagnostic difficulty, but was finally called atypical AML with vacuolated promonocytes, though in the peripheral blood we found mainly myeloblast-looking cells.

Cytocentrifuge preparations of leucocytes from 3 cases of AMML, 1 of ACMML, and 1 of smouldering AML, enabled us to identify HEA-RFC by staining the central leucocyte in the rosette. RFC found in the peripheral blood of these patients were either myeloblastic or monoblastic (including promonocytes) in appearance. There is no doubt that other cells may also form HEA rosettes, particularly those of the myelocyte series, as indicated by the smouldering AML. However, the results in essence show that the HEA-RFC in acute myeloid leukaemia are rather characteristic of the myelomonocytic leukaemias, as well as the pure monoblastic leukaemias as observed by Huber *et al.* (1969).

It is interesting that the present assay failed to detect the Fc receptors on CLL, ALL or cell lines. The results for CLL agree with those of Frøland *et al.* (1974*b*), but differ from Dickler *et al.* (1973), who used aggregated-Ig-binding as their assay for Fc receptors (Dickler and Kunkel, 1972) and found that most of the CLL patients had a high percentage of surface $Ig^+$ aggregated-Ig-binding cells. Frøland and Natvig (1973) who have separated HEA-RFC from B lymphocytes, suggested that HEA-RFC are different from the aggregated IgG-binding B cells demonstrated by Dickler and Kunkel (1972). Differences in the abilty of aggregated Ig and antibody-sensitized erythrocytes to detect Fc receptor cells might explain why Brown *et al.* (1974) failed to find any significant aggregated Ig-binding by leukaemic cells of myeloid origin, whilst we have demonstrated quite significant numbers of HEA-RFC, particularly in the AMML patients.

The demonstration of Fc receptors on AML cells is complicated by " blocking factors " whose removal enhances rosetting. Using enzyme concentrations which had relatively little effect on rosetting by normal lymphocytes (except papain), conspicuous increases in HEA-RFC were seen in a number of (though not all) AML leucocyte preparations, particularly when treated with pronase. Unsensitized HE showed no tendency to bind to enzyme-treated AML cells, neither could rosetting be detected by cell lines treated with proteolytic enzymes. The results show that pronase treatment removes " blocking factor " when it occurs, and destroys Fc receptors where the blocking factor does not occur. The pronase sensitivity of lymphocyte Fc receptors was noted by Dickler (1974) who suggested that Fc receptors *per se*, or their attachment to the cell surface, are protein or glycoprotein in nature.

HEA rosette formation by AML and normal lymphocytes was strongly inhibited by normal AB and AML serum and by Cohn Fraction II human $\gamma$-globulin, but less or not at all by Cohn Fraction V human albumin. This evidence shows that " blocking factor " is found both in normal and AML sera, and may be Ig, though whether it is complexed with an antigen is not yet known. Several authors have shown that Fc receptors on monocytes or lymphocytes can be blocked by homologous Ig, its sub-classes or fragments produced by enzyme digestion. Thus, Huber and Fudenberg (1968) found that pooled IgG or IgG1 or IgG3 had a strongly inhibitory effect on monocyte phagocytosis, whereas IgG2 and IgG4 were less effective. Very similar results were obtained by Abramson *et al.* (1970*b*), who showed in addition that Fc fragments and $\gamma$-1 and $\gamma$-3 H-chains inhibited rosetting by leucocytes, whilst $\gamma$-4 H-chain was less inhibitory, and Fab, $F(ab')_2$ and albumin were almost non-inhibitory. Other authors (Messner and Jelinek, 1970, Frøland *et al.*, 1974*a*) have observed similar inhibition of HEA-RFC, but Dickler and Kunkel (1972) were unable to inhibit aggregated Ig binding of normal lymphocytes by pre-incubation with IgG, further evidence for a difference between the HEA and aggregated Ig-binding assays for Fc receptors.

The removal of " blocking factor " by pronase treatment was found to increase the proportions of $RFC^+Ig^+$, or $RFC^+Ig^-$ cells and decrease the proportion of $RFC^-Ig^+$ cells. This could be explained by the removal of IgG by pronase digestion leading to the exposure of Fc receptors. If this is the case, then the Ig-blocking Fc receptors on AML leucocytes must be tightly bound, since they are not removed by normal cell-washing procedures. The presence of characteristic surface Ig on AML cells has previously been documented by Gutterman *et al.* (1973). It is not yet clear whether this Ig consists entirely of normal Ig aggregates or immune complexes, perhaps including viral antigens, and what proportion of it is bound to Fc receptors. There is also a possibility that some of the Fc receptor-bound Ig shows antibody activity to Fc receptors similar to that capable of inhibiting aggregated Ig-binding by B lymphocyte receptors in mice (Dickler and Sachs, 1974).

This study has shown that cells identified morphologically as myeloblasts and monoblasts have Fc receptors, though we conclude that myeloblasts in patients with acute myeloblastic leukaemia may not be as efficient in rosetting as myeloblasts in AMML patients. The myeloblasts in acute myeloblastic and acute myelomonocytic leukaemia may thus differ in their expression or exposure of Fc receptors as identified in the HEA-RFC assay. Nevertheless, the good correlation of the results with the bone marrow diagnosis supports the contention that the HEA-RFC assay could be used to diagnose AMML cases using peripheral blood leucocytes.

We thank the Medical Research Council, Leukaemia Research Fund and the Research Grants Committee of the Manchester Area Health Authority for financial support. We are indebted to our colleagues in the Department of Clinical Haematology at the Manchester Royal Infirmary for allowing us to study their patients, and to Dr C. Geary for details of the bone marrow diagnoses. We are grateful to Mrs Ann Jones, who typed the manuscript.

### REFERENCES

Abramson, N., Gelfand, E. W., Jandl, J. H. & Rosen F. S. (1970*b*). The Interaction between Human Monocytes and Red Cells. Specificity for IgG Subclasses and IgG Fragments. *J. exp. Med.*, **132,** 1207.

Abramson, N., LoBuglio, A. F., Jandl, J. H. & Cotran, R. S. (1970*a*) The Interaction between Human Monocytes and Red Cells. Binding Characteristics. *J. exp. Med.*, **132,** 1191.

Brain, P. & Marston, R. H. (1973) Rosette Formation by Human T and B Lymphocytes. *Eur. J. Immunol.*, **3,** 6.

Brown, G., Greaves, M. F., Lister, T. A., Rapson, N. & Papamichael, M. (1974) Expression of Human T and B Lymphocyte Cell-surface Markers on Leukaemic Cells. *Lancet*, ii, 753.

Böyum, A. (1968) Separation of Leucocytes from Blood and Bone Marrow. *Scand. J. clin. Lab. Invest.*, **21,** suppl. 97.

Dickler, H. B. (1974) Studies of the Human Lymphocyte Receptor for Heat-aggregated or Antigen-complexed Immunoglobulin. *J. exp. Med.*, **140,** 508.

Dickler, H. B. & Kunkel, H. G. (1972) Interaction of Aggregated $\gamma$-globulin with B Lymphocytes. *J. exp. Med.*, **136,** 191.

Dickler, H. B., Siegal, F. P., Bentwich, Z. H. & Kunkel, H. G. (1973) Lymphocyte Binding of Aggregated IgG and Surface Ig Staining in Chronic Lymphocytic Leukaemia. *Clin. exp. Immunol.*, **14,** 97.

Dickler, H. B. & Sachs, D. H. (1974) Evidence for Identity or Close Association of the Fc Receptor of B Lymphocytes and Alloantigens Determined by the Ir Region of the H-2 Complex. *J. exp. Med.*, **140,** 779.

Frøland, S. S., Michaelsen, T. E., Wisløff, F. & Natvig, J. B. (1974*a*) Specificity of Receptors for IgG on Human Lymphocyte-like Cells. *Scand. J. Immunol.*, **3,** 509.

FRØLAND, S. S. & NATVIG, J. B. (1973) Identification of Three Different Human Lymphocyte Populations by Surface Markers. *Transplant Rev.*, **16**, 114.

FRØLAND, S. S., WISLØFF, F. & MICHAELSEN, T. E. (1974*b*). Human Lymphocytes with Receptors for IgG. A Population of Cells Distinct from T and B-Lymphocytes. *Int. Archs Allergy*, **47**, 124.

GRUBB, R. (1970) The Genetic Markers of Human Immunoglobulins. In *Molecular Biology*. Biochemistry and Biophysics series No. 9. London: Chapman and Hall, Ltd.

GUTTERMAN, J. U., ROSSEN, R. D., BUTLER, W. T., MCCREDIE, K. B., BODEY, G. P., FREIREICH, E. J. & HERSH, E. M. (1973) Immunoglobulin on Tumour Cells and Tumour-induced Lymphocyte Blastogenesis in Human Acute Leukaemia. *New Engl. J. Med.*, **288**, 169.

HUBER, H., DOUGLAS, S. D. & FUDENBERG, H. H. (1969) The IgG Receptor: an Immunological Marker for the Characterisation of Mononuclear Cells. *Immunology*, **17**, 7.

HUBER, H. & FUDENBERG, H. H. (1968) Receptor Sites of Human Monocytes for IgG. *Int. Archs Allergy*, **34**, 18.

MÅRTENSSON, L. & KUNKEL, H. G. (1965) Distribution among the $\gamma$-globulin Molecules of Different Genetically Determined Antigenic Specificites of the Gm System. *J. exp. Med.*, **122**, 799.

MESSNER, R. F. & JELINEK, J. (1970) Receptors for Human $\gamma$-globulin on Human Neutrophils. *J. clin. Invest.*, **49**, 2165.

ROCHNA, E. & HUGHES-JONES, N. C. (1965) The Use of Purified $^{125}$I-labelled Anti-$\gamma$-globulin in the Determination of the Number of D Antigen Sites on Red Cells of Different Phenotypes. *Vox Sang.*, **10**, 675.

SHELDON, P. J. & HOLBORROW, E. J. (1975) Human Erythrocyte Rosette Formation with Mitogen-stimulated Human Lymphocytes—A Marker for the Demonstration of Activated T-cells. *J. Immunol., Meth.*, **7**, 379.

TERRY, W. D., FAHEY, J. L. & STEINBERG, A. G. (1965) Gm and Inv. Factors in Subclasses of Human IgG. *J. exp. Med.*, **122**, 1087.

WISLØFF, F., FRØLAND, S. S. & MICHAELSEN, T. E. (1974) Antibody-dependent Cytotoxicity Mediated by Human Fc-receptor-bearing Cells Lacking Markers for B- and T-lymphocytes. *Int. Archs Allergy*, **47**, 139.